# Survival impact of immediate complete lymph node dissection for Chinese acral and cutaneous melanoma with micrometastasis in sentinel nodes: a retrospective study

**DOI:** 10.1007/s10238-023-01107-z

**Published:** 2023-06-10

**Authors:** Jingqin Zhong, Zijian Zou, Tu Hu, Wei Sun, Chunmeng Wang, Wangjun Yan, Zhiguo Luo, Xin Liu, Yu Xu, Yong Chen

**Affiliations:** 1 Department of Musculoskeletal Surgery, Fudan University Shanghai Cancer Center, Shanghai, China; 2 Department of Medical Oncology, Fudan University Shanghai Cancer Center, Shanghai, China

**Keywords:** Acral melanoma, Cutaneous melanoma, Complete lymph node dissection, Sentinel lymph node biopsy

## Abstract

Sentinel node biopsy (SNB) has become a critical part of standard surgical treatment for melanoma with no clinical metastatic evidence. However, for patients with a positive sentinel node, the MSLT-II and DeCOG-SLT trials have shown that immediate complete lymph node dissection (CLND) does not bring further survival benefits. There is still an argument among the Chinese population dominated by acral subtypes on whether CLND can be omitted. Thus, this study aims to investigate the impact of immediate CLND on relapse-free survival (RFS) in Chinese melanoma patients with a positive sentinel node. Patients with acral or cutaneous melanoma of clinical Stages I–II who received SNB procedure and were detected with nodal micrometastasis were retrospectively collected at Fudan University Cancer Center (FUSCC) from January 2017 to December 2021. The clinicopathologic features and prognostic factors for RFS were analyzed. Out of 381 patients who received SNB in the past 5 years, 130 (34%) cases with SN micrometastasis detected were included in this study. Ninety-nine patients underwent immediate CLND while the other 31 patients received observation alone. Among patients who received CLND, the non-SN(NSN)-positive rate was 22.2%. Most of the clinicopathologic factors were balanced well between the CLND and non-CLND groups. However, more patients in the CLND group were detected with BRAF and NRAS mutation (*P* = 0.006) and received adjuvant PD-1 monotherapy (*P* = 0.042) as well. There were slightly fewer N1 patients in the CLND group, although the difference did not reach statistical significance (*P* = 0.075). The study found no significant difference in RFS between the two groups (*P* = 0.184). Even for patients with the acral subtype (*P* = 0.925), primary T4 lesion (*P* = 0.769), or presence of ulceration (*P* = 0.249), immediate CLND did not bring more survival benefits. Immediate CLND did not bring further RFS benefit for Chinese melanoma patients with SN micrometastasis in real-world clinical practice, even for patients with acral subtype or more tumor burden such as thick Breslow invasion and ulceration.

## Introduction

Sentinel node biopsy (SNB) has emerged as an indispensable element of the standard surgical procedures for Stages I–II melanoma lacking clinical metastatic evidence. The MSLT-I trial, after a decade-long monitoring, demonstrated that, although SNB failed to enhance the melanoma-specific survival (MSS) for patients with Breslow thickness above 1.2 mm compared to observation alone, it did furnish more accurate N stage to patients and could confer survival benefits to those already harboring nodal micrometastasis [1]. This has been corroborated by both the MSLT-II and DeCOG-SLT trials, which indicated that, for patients with positive sentinel node, immediate complete lymph node dissection (CLND) yielded no advantage over observation alone in terms of disease-free survival (DFS), distant metastasis-free survival (DMFS), or overall survival (OS)/MSS [2, 3]. Consequently, in multiple clinical guidelines, both CLND and observation followed by adjuvant therapy are optional for Stage III melanoma with nodal micrometastasis detected by SNB [4–6].

Nevertheless, controversy still exists among the Chinese population, particularly with regard to acral subtypes, whether CLND can be excluded, given that our patients are typically diagnosed with more aggressive disease presenting thick and ulcerated primary lesion, and high rate of SN positivity [7]. Our previous retrospective study indicated that non-sentinel node (NSN) status remained an independent prognostic factor for Chinese acral and cutaneous melanoma.

Consequently, in the absence of prospective data, we embarked on this retrospective study to explore the impact of immediate CLND on relapse-free survival (RFS) in Chinese melanoma patients with a positive sentinel node.

## Material and methods

The Ethics Committee of FUSCC granted approval for this study, and all participants consented to the operation procedures as well as their medical information collection by signing informed consent documents.

### Patient selection

Our retrospective study involved the recruitment of patients with acral or cutaneous melanoma of clinical Stages I–II, who underwent SNB procedure and were subsequently diagnosed with nodal micrometastasis at Fudan University Cancer Center (FUSCC) during the period between January 2017 and December 2021. Patients with incomplete medical information or who were followed up for a period shorter than 6 months were excluded. Clinicopathological characteristics including gender, age, Breslow thickness, presence of ulceration, SN and NSN status, metastatic burden in SN, as well as adjuvant therapy and follow-up information were obtained from the patient database in FUSCC.

Our retrospective study involved the recruitment of patients with acral or cutaneous melanoma of clinical Stages I–II, who underwent sentinel node biopsy (SNB) and were subsequently diagnosed with nodal micrometastasis at Fudan University Cancer Center (FUSCC) during the period between January 2017 and December 2021. Patients with incomplete medical information or who were followed up for a period shorter than 6 months were excluded. Gender, age, Breslow thickness, presence of ulceration, sentinel node (SN) and non-sentinel node (NSN) status, metastatic burden in SN, as well as adjuvant therapy and follow-up information were obtained from the patient database at FUSCC.

### Operative procedures

Lymphatic mapping with a combination of lymphoscintigraphy using technetium-99 sulfur colloid and methylene blue dyeing was used to identify SNs in each patient. The pathological assessment of each SN resected involved both hematoxylin and eosin (HE) staining, as well as immunohistochemistry of S-100, HMB45, Melan-A, and SOX10 on paraffin-embedded specimen sections.

The decision on whether to proceed with immediate CLND, as well as choice of adjuvant therapy, was made based on recommendations from physicians and the patients' preferences. All CLND procedures were completed within 1 month after SNB and were only performed in cases where the SN was found to be positive. All NSN specimens were evaluated using routine HE staining.

### Follow-up information

All patients were monitored through clinical examination and imaging assessment, including ultrasound, CT/MRI scans, every 3 months for the first 2 years, every 6 months for 3–5 years, and then annually after the surgery. Relapse and survival information was obtained from either outpatient visits or telephone follow-ups.

Local relapse or regional relapse was defined as the recurrence of the primary lesion, in-transit disease, or in the regional lymph node basin. Systemic relapse was defined as the occurrence of distant metastasis. Either imaging or pathology was used to confirm the recurrence or metastasis. RFS was defined as the time interval between the SNB and the first occurrence of local and regional relapse or distant metastasis.

### Statistical analysis

Pearson’s Chi-squared test or Fisher’s exact test was used to perform univariable analyses of clinicopathological factors between different category groups. Kaplan–Meier estimations and log-rank tests were used to identify prognostic factors for RFS. *P*-values less than 0.05 were considered statistically significant. All statistical analyses were performed using SPSS software (version 22.0).

## Results

### Patients’ characteristics

Out of 381 patients who underwent SNB in the past 5 years, 130 cases (34.1%) were identified as having micrometastasis in the SN and were recruited for our study.

Out of the 130 patients who were recruited for our study, 85 (65.4%) had acral melanoma, while the other 45 (34.6%) had cutaneous subtype. Among the patients, 67 (41.5%) were male, with a median age of 59 years old. The mean Breslow thickness was determined to be 3.4 mm, and the ulceration rate was 60.0%. The most common gene mutations found were BRAF (32.3%), NRAS (16.2%), and CKIT (9.2%). Adjuvant treatment options included anti-PD1 monotherapy for 73 (56.1%) patients, targeted therapy using BRAF inhibitor alone or in combination with MEK inhibitor for 17 (13.1%) patients, interferon for 15 (11.5%) patients, and observation alone for the remaining 25 (19.2%) patients.

### SNB and CLND

Out of the 130 recruited patients, 98 (75.4%) received SNB in groin basin, while 27 (20.8%) received SNB in axilla basin. The median number of SN biopsied was 2. About 70.7% (92/130) of patients had only one positive SN. Among the 89 patients whose tumor burden was assessed in the SN, 39 (42.8%) had maximum diameter (*D*_max_) of micrometastasis less than 1 mm.

Ninety-nine patients underwent immediate CLND while the remaining 31 received observation alone. Among the patients who underwent CLND, the rate of NSN positivity was 22.2%.

Table 1 compares various clinicopathologic factors between patients who underwent immediate CLND and those who did not. The analysis showed that there was no significant difference in gender, age, subtype, SNB basin, primary thickness (Breslow), presence of ulceration, number of positive SN, number of SN biopsied, or *D*_max_ of SN between the two groups. However, there were a slightly lower proportion of N1 stage in CLND group compared to no CLND group although the difference did not reach statistical significance (*P* = 0.075). Meanwhile, the proportion of patients with BRAF and NRAS mutations (*P* = 0.006) and those who received adjuvant anti-PD1 treatment (*P* = 0.042) was significantly higher in the CLND group.

**Table 1:** Clinicopathological characteristics categorized in patients with or without CLND

	No CLND (%) *n* = 31	CLND (%) *n* = 99	*P*-value
*Gender*			0.687
Female	16 (51.6%)	47 (47.5%)	
Male	15 (48.4%)	52 (52.5%)	
*Age*			0.093
< 60 years	10 (32.3%)	49 (49.5%)	
≥ 60 years	21 (67.7%)	50 (50.5%)	
*Subtype*			0.752
Acral	21 (67.7%)	64 (64.6%)	
Cutaneous	10 (32.3%)	35 (35.4%)	
*SNB basin*			0.244
Axilla	7 (22.6%)	20 (20.2%)	
Groin	21 (67.7%)	77 (77.8%)	
Multi-basin	3 (9.7%)	2 (2.0%)	
*Breslow thickness (T stage)*			0.516
0–1 mm	2 (6.5%)	7 (7.1%)	
1–2 mm	7 (22.6%)	16 (16.2%)	
2–4 mm	13 (41.9%)	33 (33.3%)	
> 4 mm	9 (29.0%)	43 (43.4%)	
*Ulceration*			0.801
No	13 (41.9%)	39 (39.4%)	
Yes	18 (58.1%)	60 (60.6%)	
*Number of SN positive*			0.778
1	23 (74.2%)	69 (69.7%)	
> 1	8 (25.8%)	30 (30.3%)	
*Number of SN biopsied*			0.788
1–2	17 (54.8%)	57 (57.6%)	
> 2	14 (45.2%)	42 (42.4%)	
*D*_*max*_			0.468
< 1 mm	12 (38.7%)	27 (27.3%)	
≥ 1 mm	10 (32.3%)	40 (40.4%)	
*NSN status*			
Negative	–	77 (77.8%)	
Positive	–	22 (22.2%)	
*N stage*			0.075
N1	23 (74.2%)	52 (52.5%)	
N2	8 (25.8%)	42 (42.4%)	
N3	0	5 (5.1%)	
*Gene mutation*			0.006*
BRAF	8 (25.8%)	34 (34.3%)	
NRAS	0	16 (16.2%)	
CKIT	7 (22.6%)	5 (5.1%)	
Wild type	12 (38.7%)	37 (37.4%)	
Untested	4 (12.9%)	7 (7.1%)	
*Adjuvant therapy*			0.042*
IFN	3 (9.7%)	12 (12.1%)	
PD-1	12 (38.7%)	61 (61.6%)	
Targeted therapy	5 (16.1%)	12 (12.1%)	
Observation	11 (35.5%)	14 (14.1%)	
*Relapse mode (initial)*			0.295
LR	3 (9.7%)	21 (21.2%)	0.515
Systemic	5 (16.1%)	18 (18.2%)	
No relapse	23 (74.2%)	60 (60.6%)	

*CLND* complete lymph node dissection, *SN* sentinel node, *SNB* sentinel node biopsy, *D*_*max*_ maximum diameter of SN micrometastasis, *NSN* non-sentinel node, and *LR* local and regional

*Statistically significant

### Relapse-free survival

As of the cutoff date of December 31, 2021, the median follow-up time of the no CLND and CLND group were 24 months and 21 months, respectively.

**Fig. 1:**
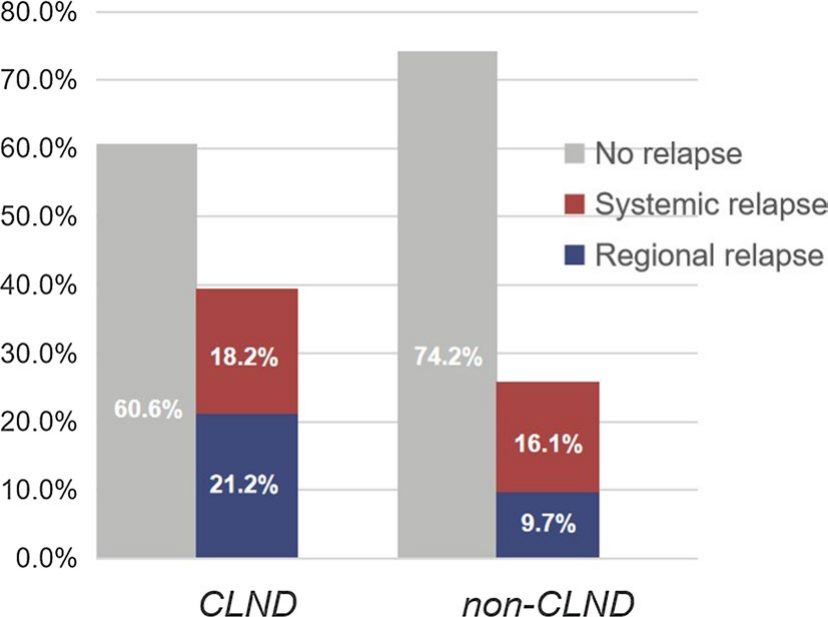
Proportion of recurrence patterns in the group of CLND and non-CLND

A total of 8 (25.8%) patients in the no CLND group and 39 (39.4%) in the CLND group experienced relapse. Surprisingly, the CLND group had a higher rate of local and regional relapse (21.2%) compared to the no CLND group (9.7%), but the rate of systemic relapse was comparable between two groups (18.2% vs 16.1%) (Fig. 1).

**Fig. 2:**
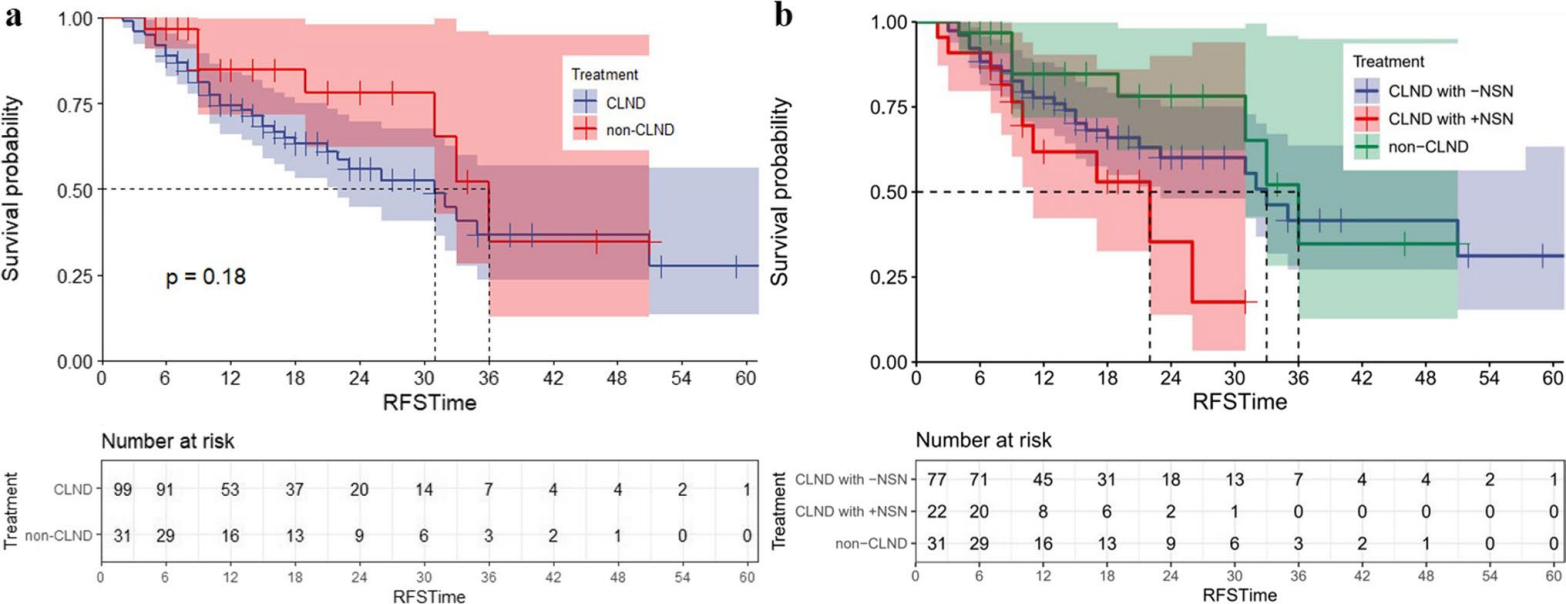
RFS curve for all patients. **a** non-CLND vs CLND and **b** non-CLND vs CLND with negative NSN vs CLND with positive NSN

There was no significant difference in RFS between the two groups [median RFS (mRFS), non-CLND vs CLND: 36 months vs 31 months, *P* = 0.184, Fig. 2a]. The 1-year and 2-year RFS rates of the non-CLND group were 84.7% and 78.2%, respectively, while those of the CLND group were 74.5% and 55.9%, respectively.

Compared to the no CLND group, patients in the CLND group who had negative NSN had a comparable RFS (mRFS 33 months, *P* = 0.330). However, patients in the CLND group who had positive NSN experienced significant worse RFS (mRFS 22 months, *P* = 0.018) (Fig. 2b).

**Fig. 3:**
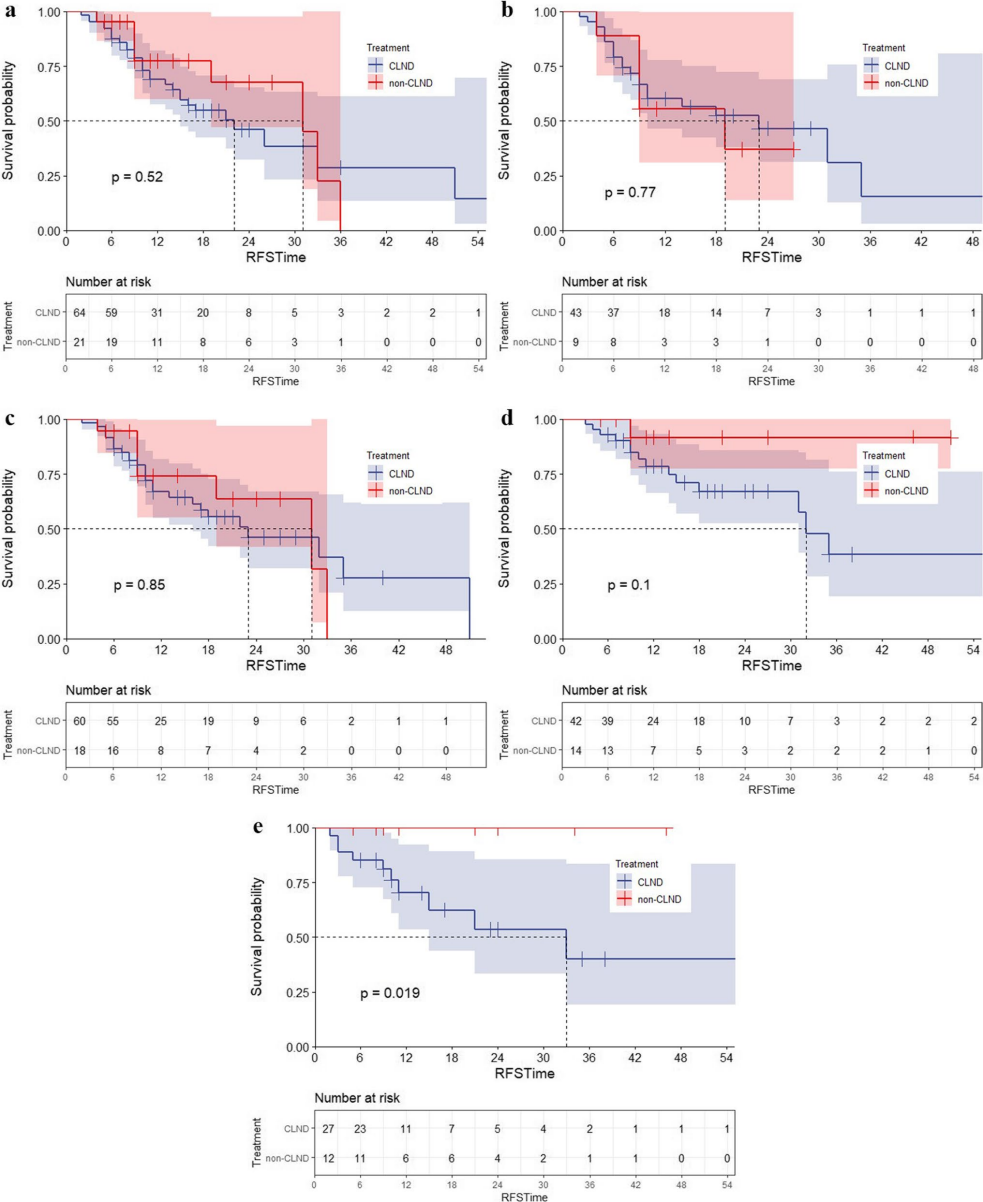
RFS curve for patients in subgroup. **a** RFS curve for patients with melanoma of acral subtype, **b** RFS curve for patients with T4 melanoma, **c** RFS curve for patients with melanoma with ulceration, **d** RFS curve for patients with melanoma with number of SN biopsied ≥ 3, and **e** RFS curve for patients with melanoma with SN *D*_max_ < 1 mm

Further subgroup analysis was conducted, and there was still no significant difference in RFS between the no CLND and CLND groups for patients with acral type (*P* = 0.520), primary T4 lesion (*P* = 0.769), or presence of ulceration (*P* = 0.850) (Fig. 3a–c). Interestingly, in patients who had more than 2 SN biopsied (*P* = 0.100) and in those who had a SN *D*_max_ < 1 mm (*P* = 0.019), the no CLND group appeared to have even better RFS than CLND group (Fig. 3d and f).

## Discussion

To the best of our knowledge, this is the first study conducted in the Chinese population to investigate whether immediate CLND could bring further survival benefit for Stage III melanoma with SN micrometastasis. Consistent with the MSLT-II and DeCOG-SLT trials, our retrospective data support the conclusion that immediate CLND might not improve RFS compared to observation alone, even for patients with acral subtype, or more tumor burden such as T4 or ulcerated primary lesion.

Previous concerns about the omission of CLND after a positive SN mainly related to the dominance of acral subtype in Chinese melanoma patients. Several studies have shown that acral melanoma tends to present with deeper Breslow thickness, a higher rate of ulceration and nodal involvement, and a more advanced stage and worse prognosis [8, 9]. Additionally, the SN- and NSN-positive rate increases with primary lesion thickness. In the MSLT-I trial, the SN-positive rate for intermediate-thickness melanomas (Breslow thickness 1.2–3.5 mm) and thick melanomas (Breslow thickness > 3.5 mm) was 16% and 32%, respectively [1]. In the MSLT-II trial, only 22% of recruited patients had a Breslow thickness of more than 3.5 mm, and the NSN-positive rate was 12% in the immediate CLND arms [2]. However, in our previous studies based on Chinese population, the mean Breslow thickness was found to be 3.6 mm. Both the SN- and NSN-positive rate were around 30%, which is significantly higher than those reported in the MSLT trials [10, 11]. Therefore, theoretically for Chinese melanoma patients, there may be a higher risk of residual nodal disease without immediate CLND, leading to an increased risk of regional recurrence in the basin as well as systemic dissemination.

However, the results from this retrospective study were not consistent with our expectations. In our melanoma cohort from the past 5 years, with a median Breslow thickness of 3.4 mm and an ulceration rate of 60%, the SN- and NSN-positive rates remained as high as 34% and 22%, respectively. However, whether patients received immediate CLND or not did not affect the RFS outcomes. Surprisingly, even for patients with acral subtype, or more aggressive primary lesion with Breslow thickness > 4 mm or ulceration, immediate CLND failed to improve RFS either. There may be several reasons for these findings. First of all, there might be a selection bias where surgeons were more likely to recommend immediate CLND for patients with higher disease burden. In our study, although clinicopathologic factors were almost balanced between no CLND and CLND groups, there were still more T4 patients underwent CLND (T4, no CLND vs CLND: 29% vs 43.4%). On the other hand, it might also be due to the progression pattern of thick melanomas. In the MSLT-I trial, SNB with following CLND did bring survival benefits in DFS and MSS for intermediate melanomas (Breslow thickness 1.2–3.5 mm), but not for thick melanomas (Breslow thickness > 3.5 mm) [1]. In DeCOG-SLT trial, similar time intervals were calculated to develop regional nodal recurrence or distant metastasis [3]. In recent published retrospective study of over 1400 invasive melanoma cases, the risk of distant metastasis had suppressed the risk of local or nodal recurrence for T4 melanoma [12]. Regarding the relapse mode in our data, the systemic relapse rate was observed similarly in no CLND and CLND group (16.1% vs 18.2%). Hence, the real reason, why immediate CLND could not bring more survival benefit, is that patients with thick melanoma are facing more danger from hematogenous rather than lymphatic metastases.

Interestingly, we found that immediate CLND might worsen outcomes for patients who received more than 2 SN biopsies or had SN micrometastasis *D*_max_ less than 1 mm. Although these findings could be biased due to the limited samples in subgroup analyses, changes in anti-tumor immunity after CLND might still be considered, especially in new era of immunotherapy. The regional lymph node basin is a crucial organ for tumor-associated antigen recognition and T-cell activation. For patients who had adequate nodes biopsied and lower metastatic burden, and less chance for residual nodes after SNB, CLND might not be able to bring any therapeutic value further, but negatively regulate immunization. However, we did not find RFS difference with or without CLND for patients receiving anti-PD1 adjuvant therapy in this study due to the small sample in each adjuvant subgroup (*P* = 0.363). So far, there is one randomized adjuvant trial, Checkmate 915, in which CLND was not mandatory for SN-positive patients recruited [14]. Although the adjuvant nivolumab treatment arm in Checkmate 915 was reported with similar 2-year RFS rates (64.7% vs 67%) to the same arm in Checkmate 238 (mandatory CLND for all patients), no data have been revealed on the SN-positive subgroup yet. Further trials should be designed to investigate the impact of CLND on adjuvant immunotherapy for patient with nodal micrometastasis.

Moreover, in our study, patients detected with positive NSN after CLND did have worse outcome compared to both NSN-negative cases and patients without CLND. It suggests that predicting NSN status might still be important in the future practice. Several studies have been published to evaluate prognostic factors or establish clinicopathologic models to predict positive NSN [15, 16]. In our previous study based on 328 SN-positive melanoma in the Chinese population, Breslow thickness, Clark level, and the number of positive SNs were independently related to positive NSN. NSN status was also an independent factor for DFS [7]. However, in a recent published paper of further analyses on SN-positive patients in the MSLT-I trial, no factor was significantly associated with NSN status, and the presence of NSN metastasis was not a significant predictor of MSS either [17]. Although factors affecting MSS could be complex than those for DFS, it might also be because of the higher proportion of acral subtypes (67.1%) and NSN-positive rate (30.2%) in our Chinese population compared to those in the MSLT-I trial (mostly cutaneous melanoma and only 11.3% NSN-positive rate). Contradictory results were found in the MSLT-II and DeCOG-SLT trials. While NSN status was reported to be a significant prognostic factor in the former, it failed to be one for DMFS, OS, and RFS in the latter. Recent efforts have been focused on investigating tumor burden of SN micrometastasis or gene expression score (GEP) of the primary tumor on their impact on predicting survival [17, 18].

Obviously, our study still has several limitations. As in any retrospective review, the results might be confounded by selection bias. Also, data from one single center restricted the patient population. Furthermore, OS data were not analyzed in our study due to the lack of enough events of death occurring in both groups within such a short time. Therefore, further investigation by prospective and multicenter research with extended follow-ups should be considered.

## Conclusion

Our retrospective study implied that immediate CLND did not bring further RFS benefit after an observation for Chinese melanoma with SN micrometastasis in real clinical practice, even for patients with the acral subtype or more tumor burden such as thick Breslow invasion and ulceration. Further prospective studies are still needed to investigate biomarkers that can efficiently predict NSN status and survival outcomes for SN-positive patients in the Chinese population.
